# Beyond the Wild MRSA: Genetic Features and Phylogenomic Review of *mecC*-Mediated Methicillin Resistance in Non-*aureus* Staphylococci and Mammaliicocci

**DOI:** 10.3390/microorganisms12010066

**Published:** 2023-12-29

**Authors:** Idris Nasir Abdullahi, Javier Latorre-Fernández, Rine Christopher Reuben, Islem Trabelsi, Carmen González-Azcona, Ameni Arfaoui, Yahaya Usman, Carmen Lozano, Myriam Zarazaga, Carmen Torres

**Affiliations:** 1 Area of Biochemistry and Molecular Biology, OneHealth-UR Research Group, University of La Rioja, 26006 Logroño, Spain; idris-nasir.abdullahi@unirioja.es (I.N.A.); jl471998@gmail.com (J.L.-F.); rine-christopher.reuben@unirioja.es (R.C.R.); carmen.gonzalezaz@unirioja.es (C.G.-A.); carmen.lozano@unirioja.es (C.L.); myriam.zarazaga@unirioja.es (M.Z.); 2 Bioresources, Environment and Biotechnology Laboratory, Higher Institute of Applied Biological Sciences of Tunis, University of Tunis El Manar, Tunis 1006, Tunisia; islem.trabelsi@etudiant-issbat.utm.tn; 3 Laboratory of Microorganisms and Active Biomolecules, Faculty of Sciences of Tunis, University of Tunis El Manar, Tunis 1068, Tunisia; arfaoui.ameni109@gmail.com; 4 Department of Medical Laboratory Science, Ahmadu Bello University, Zaria 810107, Nigeria; elyahaya98@gmail.com

**Keywords:** *Mammaliicoccus sciuri*, *Mammaliicoccus lentus*, bovine mastitis, SCC*mec-mecC* hybrid, wild MRSA

## Abstract

Methicillin resistance, mediated by the *mecA* gene in staphylococci and mammaliicocci, has caused tremendous setbacks in the use of antibiotics in human and veterinary medicine due to its high potential of presenting the multidrug resistance (MDR) phenotype. Three other *mec* analogs exist, of which the *mecC* has evolutionary been associated with methicillin-resistant *Staphylococcus aureus* (MRSA) in wild animals, thus loosely referred to as the wild MRSA. In this study, we present an epidemiological review and genomic analysis of non-*aureus* staphylococci and mammaliicocci that carry the *mecC*-mediated methicillin resistance trait and determine whether this trait has any relevant link with the One Health niches. All previous studies (2007 till 2023) that described the *mecC* gene in non-*aureus* staphylococci and mammaliicocci were obtained from bibliometric databases, reviewed, and systematically analyzed to obtain the antimicrobial resistance (AMR) and virulence determinants, mobilome, and other genetic contents. Moreover, core genome single-nucleotide polymorphism analysis was used to assess the relatedness of these strains. Of the 533 articles analyzed, only 16 studies (on livestock, environmental samples, milk bulk tanks, and wild animals) were eligible for inclusion, of which 17 genomes from 6 studies were used for various in silico genetic analyses. Findings from this systematic review show that all *mecC*-carrying non-*aureus* staphylococci were resistant to only beta-lactam antibiotics and associated with the classical SCC*mec* XI of *S. aureus* _LGA251_. Similarly, two studies on wild animals reported *mecC*-carrying *Mammaliicoccus stepanovicii* associated with SCC*mec* XI. Nevertheless, most of the *mecC*-carrying *Mammaliicoccus* species presented an MDR phenotype (including linezolid) and carried the SCC*mec*-*mecC* hybrid associated with *mecA.* The phylogenetic analysis of the 17 genomes revealed close relatedness (<20 SNPs) and potential transmission of *M. sciuri* and *M. lentus* strains in livestock farms in Algeria, Tunisia, and Brazil. Furthermore, closely related *M. sciuri* strains from Austria, Brazil, and Tunisia (<40 SNPs) were identified. This systematic review enhances our comprehension of the epidemiology and genetic organization of *mecC* within the non-*aureus* staphylococci and mammaliicocci. It could be hypothesized that the *mecC*-carrying non-*aureus* staphylococci are evolutionarily related to the wild MRSA-*mecC*. The potential implications of clonal development of a lineage of *mecA/mecC* carrying strains across multiple dairy farms in a vast geographical region with the dissemination of MDR phenotype is envisaged. It was observed that most *mecC*-carrying non-*aureus* staphylococci and mammaliicocci were reported in mastitis cases. Therefore, veterinarians and veterinary microbiology laboratories must remain vigilant regarding the potential existence of *mecA/mecC* strains originating from mastitis as a potential niche for this resistance trait.

## 1. Introduction

The genera *Staphylococcus* and *Mammaliicoccus* are predominantly nasal and skin commensals in humans and most animal species [1,2,3]. However, they could be translocated to other parts of the human and animal body to cause clinical infections through the expressions of virulence genes [4]. The antimicrobial resistance (AMR) and virulence potential of staphylococci have long been elucidated in detail in *S. aureus.* However, non-*aureus* staphylococci and *Mammaliicoccus* species have recently been shown to carry critical AMR genes and virulence factors that have been hitherto exclusively reported in *S. aureus* [5,6,7]. In these regards, it is important to remark on the detection of linezolid resistance genes (*cfr*, *optrA,* and *poxtA*) and virulence determinants such as *tst*, *luk-F/S-PV*, *eta*, *seb*, *sec*, and *sel* in some non-*aureus* staphylococci and mammaliicocci [5,6,7,8,9]. AMR is a major global health challenge that needs a holistic “One Health” approach, for which *Staphylococcus* and certain *Mammaliicoccus* species serve as suitable bacteria models. This is because some species and lineages could “spill over” across multiple hosts, carry emergent resistance mechanisms or transfer critically important AMR zoonotically or anthropogenically [10]. Recently, studies have shown enormous interrelations of the wildlife–livestock interface in the transmission and maintenance of bacterial pathogens and AMR of public health concerns such as those caused by staphylococci and, by extension, mammaliicocci [11,12,13].

The presence of methicillin resistance and resistance to nearly all beta-lactams in staphylococci were historically linked to the acquisition of the *mecA* gene, which encodes the alternative penicillin-binding protein PBP2a [14]. However, the frequent association of methicillin resistance in staphylococci, mammaliicocci, and macrococci has now been attributed to the presence of other *mec*-type genes (*mecB*, *mecC*, and *mecD*) (Table 1). These genes also encode for penicillin-binding proteins (PBPs) that exhibit low affinity for beta-lactams [15]. 

The *mecA*-mediated methicillin-resistant *S. aureus* (MRSA) exhibits a high prevalence on a global scale in human and multiple animal hosts, especially in pigs and dairy animals [10,26,27]. In 2007, an additional *mec* gene, known as *mecC*, was discovered to be associated with resistance to beta-lactam antibiotics during an epidemiological investigation of bovine mastitis [16,28]. The *mecC* gene, previously known as *mecA*_LGA251_, is a *mecA* variant that shares 69% nucleotide identity and was initially reported in an *S. aureus* strain from a bovine sample [29]. Similar to *mecA*, *mecC* was discovered to be present inside the mobile genetic element (MGE) referred to as the staphylococcal cassette chromosome *mec* (SCC*mec*), which is inserted at the 3′ end of the *orfX* locus [29]. 

The SCC*mec* harboring *mecC* exhibited notable distinctions from previously identified types and was officially classified as SCC*mec* type XI [30,31]. In addition to its presence in cattle, *mecC* has been documented in MRSA strains from people throughout various European countries, as well as in a wide range of wild animal species as reviewed by Abdullahi et al. [27] and Lozano et al. [32]. Furthermore, *mecC*-carrying MRSA strains have also been demonstrated in river water and livestock such as sheep and goats in Spain and Tunisia [33].

The *mecC* allotype was subsequently discovered in *Mammaliicoccus* (previously *Staphylococcus*) *sciuri*, located downstream of the newly identified SCC*mec* type VII [34]. This hybrid SCC*mec-mecC* element consists of *mecA* and *mecC* regions organized within a class E *mec* complex (*mecI-mecR*, *mecC-blaZ*) [34]. It has been demonstrated a strong correlation between *M. sciuri* and the origin and assembly of the SCC*mec* element, especially for SCC*mec* type III [35]. Consequently, several previous investigations have shown multiple lines of evidence indicating that the *mecA1* gene originated in *M. sciuri* encoding the PBPD [36]. Furthermore, Rolo et al. [35] provided evidence that *M. sciuri* species serve as an innate host and abundant reservoir for *ccr*, which is the most likely source of these recombinases for the formation of SCC*mec* [15]. Nevertheless, there are limited data regarding the origin and molecular epidemiology and the clinical importance of *mecC*-carrying mammaliicoccal strains. So far, *M. sciuri* has been found in environmental and animal samples [8,37,38] and has been associated with occasional infections in both animals and humans [39,40,41,42]. Previous research has demonstrated that mammaliicocci strains bearing *mecA/mecC* homologs exhibit the ability to excise both the hybrid SCC*mec-mecC* and SCC*mec* type XI from the chromosome [34]. Furthermore, these elements can be subsequently transmitted to more pathogenic staphylococci [43]. In this study, we present an epidemiological review and molecular analysis of non-*aureus* staphylococci and mammaliicocci that carry the *mecC*-mediated methicillin resistance trait and determine whether this trait has any relevant link with the One Health niches.

## 2. Methodology

### 2.1. Literature Search

A comprehensive literature review was conducted on the PubMed database using the following search terms: “methicillin”, “*mecC* CoNS”, “*mecC* methicillin”, “*mecC*-methicllin resistance”, “*mecC* mammaliicocci”, “*mecC* mastitis”, “*mecC* livestock”, “*mecC* dairy”, “*mecC* environment”, “*mecC* wild animal”, “*mecC S. sciuri*”, “*mecC* non-*aureus*”, “*mecC* human”, “*mecC* dog”, and “*mecC* cat”. Additional search engines such as Google Scholar, ScienceDirect, Scopus, and Web of Science were used to obtain all potentially eligible studies. The inclusion criteria encompassed articles that were published throughout the time frame of October 2007 to October 2023. Of the 533 hits, a total of 341 articles were removed, as they did not address non-*aureus* staphylococci, as indicated in Supplementary Figure S1. An additional 176 articles were omitted from the study, as they solely concentrated on the *mecA*-mediated methicillin resistance, inadequate methodology, or review papers. An evaluation was conducted on 16 studies that specifically examined *mecC*-carrying non-*aureus* staphylococci and mammaliicocci (Table 2). From these 16 articles, only 6 met the criteria for detailed genomic analyses, as indicated in Supplementary Figure S1 and Table 3.

### 2.2. Description of the mecC-Carrying Non-aureus Staphylococci and Mammaliicocci Strains and the Methodology Used in the Eligible Studies

The strains included in this analysis and obtained from the eligible studies (Table 2), encompassed a diverse range of subjects, including livestock suffering from mastitis, as well as specimens obtained from farms and wild animals. Non-*aureus* staphylococci and mammaliicocci from the eligible studies were obtained from several sources, including milk, teat, manure, soil, and skin samples. Following the collection of samples in these studies, they were subjected to cultivation; subsequently, their DNA was extracted for various gene amplifications, and whole-genome sequencing (in some studies). The disc diffusion method was commonly utilized in most studies to assess resistance to oxacillin and/or cefoxitin in antibiotic susceptibility tests. The genomic sequences were utilized to identify the mechanisms for methicillin resistance and other AMRs. Additionally, the genomes of the strains obtained from GenBank were used to determine the sequence types (STs), virulome, plasmids, SCC*mec* types, and other MGEs (Table 2). 

### 2.3. Phylogenetic and In Silico Genomic Analysis 

To determine the relatedness of the non-*aureus* staphylococci and mammaliicocci strains from the eligible studies, a web-based CSI phylogeny database (https://cge.food.dtu.dk/services/CSIPhylogeny/) (accessed on 10 September 2023) was used to obtain the SNPs by mapping the publicly available genomes of the 17 strains obtained from GenBank to a reference *S. aureus* _LGA251_ (accession number FR821779) with the default parameter, except for that the minimum distance between SNPs, which was disabled. The graphical data were added to the phylogenies using iTOL v.6.6 [44]. The sequence types (STs) were determined using MLST v.2.16 [45]. Virulence factors, plasmid replicons, and antimicrobial resistance genes were identified using PlasmidFinder, and Resfinder from the Center for Genomic Epidemiology. Moreover, other databases such as VFDB (http://www.mgc.ac.cn/VFs/main.htm (accessed on 12 September 2023) and CARD (https://card.mcmaster.ca/analyze/rgi) (accessed on 12 September 2023) were used to search for additional virulence and AMR genes. The genetic environment of the *mecC* gene from 10 non-*aureus* staphylococci and mammaliicocci strains (one per species per study) was illustrated in comparison with the *S. aureus* _LGA251_ strain (accession number FR821779). Computations and graphical designs were performed using EasyFig (https://mjsull.github.io/Easyfig/) (accessed on 28 October 2023) and Inkscape software version 1.3.2. (https://inkscape.org/) (accessed on 28 October 2023).

**Table 2:** AMR, virulence genes, genetic lineages, and mobile genetic elements in *S. aureus*_LGA251_, and in *mecC*-carrying non-*aureus* staphylococci and mammaliicocci.

Authors	Country	Source of the Strains	Bacterial Species (Number)	AMR Phenotype	Molecular Assays	AMR Genes	Plasmid Reps (Associated AMR)	Genetic Lineage	SCC*mec* Type	Other MGEs
García-Álvarez et al. [16]	UK	Bulk milk	*S. aureus* (1)	PEN, OXA	WGS	*blaZ*, *mecC*	ND	ST425	XI	None
Harrison et al. [22]	UK	Bovine milk	*S. xylosus* (1)	PEN, OXA	WGS	*blaZ*, *mecC*	NT	NT	XI	Tn*554*-like
MacFadyen et al. [46]	UK	Bulk milk tank	*S. xylosus* (1)	PEN, OXA	WGS	*blaZ*, *mecC*	NT	NT	XI	ACME
Paterson et al. [40]	UK	Bovine milk tank	*M. sciuri* (11)	PEN, OXA, CLI, TET, STR	WGS	*blaZ*, *mecC*, *salA*, *tet*(K), *str*	NT	NA	SCC*mec-mecC* hybrid	None
Harrison et al. [34]	UK	Bovine	*M. sciuri* (2)	PEN, OXA, FOX, CHL, CLI, TET, STR, FUS	*WGS*	*blaZ*, *mecA*, *mecA1*, *mecC*, *fexA*, *ermC*, *tet*(K), *str*	NT	NA	SCC*mec-mecC* hybrid	None
Dhaouad et al. [39]	Tunisia	Calves, cow, horses, rabbit	*M. sciuri* (6)	PEN, FOX, CHL, ERY, CLI, GEN, TOB, STR, TET, FUS	WGS	*blaZ*, *mecA*, *mecA1*, *mecC*, *fexA*, *erm45*, *ermB*, *salA*, *aac6′-aph2″*, *ant4*, *str*, *dfrK*, *tet*(K), *tet*(L), *fusB/C*	*rep22* (*ant4′*, *dfrK*, *tet*(L)), *repUS76* (*ermB*)	ST38	SCC*mec-mecC* hybrid	Tn*558* (*fexA*)
de Moura et al. [47]	Brazil	Bovine	*M. sciuri* (2)	PEN, FOX, CLI, TET, STR	WGS	*blaZ*, *mecA*, *mecA1*, *mecC*, *salA*, *str*, *tet*(K)	*rep7a* (*str*)	ST71	SCC*mec-mecC* hybrid	None
Aslantaş [48]	Turkey	Broilers	*M. sciuri* (7)	PEN, FOX, ERY, CLI, TET, GEN, SXT	PCR	*blaZ*, *mecA*, *mecC*, *ermA*, *lnuA*, *tet*(K), *tetM*, *aac6-aph2*	NT	NT	III (by PCR)	NT
Belhout et al. [49]	Algeria	Camels	*M. lentus* (5)	PEN, FOX, STR, ERY, CLI, TET	WGS	*blaZ*, *mecA*, *mecC*, *str*, *ermB*, *mphC*, *tet*(K)	*rep7a* (*tet*(K), *str*)	ND	SCC*mec-mecC* hybrid	None
Srednik et al. [50]	Argentina	Bovine	*S. saprophyticus* (1)	PE, OXA, FOX	PCR	*blaZ*, *mecC*	NT	NT	NT	NT
Małyszko et al. [23]	Poland	Shrew (small mammal)	*S. saprophyticus* (1)	PEN, OXA	PCR	*blaZ*, *mecC*	NT	NT	NT	NT
Loncaric et al. [51]	Austria	Eurasian lynx	*M. stepanovicii* (1)	PEN, OXA	PCR	*blaZ*, *mecC*	NT	NT	NT	NT
Semmler et al. [52]	Germany	Wild vole	*M. stepanovicii* (1)	PEN, OXA	WGS	*blaZ*, *mecC*	NT	ND	XI	None
Lancoric et al. [53]	Austria	Wild and domestic animals	*M. stepanovicii*, *S. caprae*, *S. warneri*, *S. xylosus*, and *M. sciuri*	a. *M. sciuri* (PEN, OXA, FOX, GEN, TET, ERY, CLI, CHL, SXT) b. *M. stepanovicii*, *S. caprae*, *S. xylosus*, *S. warneri* (PEN, FOX)	PCR, WGS	a. *blaZ*, *mecA*, *mecA1*, *mecC ant4′*, *tet*(M), *ermB*, *cfr*, *fexA* in *M. sciuri* *b. blaZ*, *mecC* in others	ND	*M. sciuri* (ST22)	a. SCC*mec-mecC* hybrid in *M. sciuri* b. XI in others	None
Pantůček et al. [54]	The Czech Republic	Stone fragments/sandy soil	*S. edaphicus sp. nov.* (1)	PEN, OXA	WGS	*blaZ*, *mecC*	NT	ND	XI	None
Dhaouad et al. [38]	Tunisia	Bovine mastitis and manure	*M. sciuri*	PEN, OXA, FOX, TET	PCR	*mecA*, *mecC*, *blaZ*, *tet*(K)	NT	NT	Non-typeable	NT
Abdullahi et al. [37]	Spain	Nestling of white stork	*M. lentus*	PEN, FOX, CLI, TET	PCR	*blaZ*, *mecA*, *mecC*, *mphC*, *tet(K)*	NT	NT	*blaZ*-SCC*mec* XI	NT

**Abbreviations:** PCR: polymerase chain reaction; WGS: whole-genome sequencing; NT: not tested; NA: not applicable; ST: sequence type: CLI: clindamycin; CHL: chloramphenicol; CIP: ciprofloxacin; ERY: erythromycin; FOX: cefoxitin; FUS: fusidic acid; GEN: gentamicin; OXA: oxacillin; PEN: penicillin; TET: tetracycline; TOB: tobramycin; STR: streptomycin; SXT: sulfamethoxazole–trimethoprim.

**Table 3:** Species and sources of genomes used for the phylogenomic analyses in this review.

Authors	Country	Strain	GenBank Accession Number
García-Álvarez et al. [16]	UK	*S. aureus*_LGA251_	FR821779
Dhaouad et al. [39]	Tunisia	*M. sciuri*	SRR20693405 SRR20693403 SRR20693382 SRR20693383 SRR20693384
Paterson et al. [40]	UK	*M. sciuri*	ERR3350388
Lancoric et al. [53]	Austria	*S. xylosus* *S. warneri* *M. scuiri*	SRR8494495 SRR8494496 SRR8494497
Pantůček et al. [54]	The Czech Republic	*S. edaphicus*	GCA 002614725
de Moura et al. [47]	Brazil	*M. sciuri*	GCA 030250115.1 GCA 030250065.1
Belhout et al. [49]	Algeria	*M. lentus*	GCA 030013965.1 GCA 030012945.1 GCA 030012925.1 GCA 030012985.1

## 3. Findings and Discussion

### 3.1. SCCmec and Its Classification System in Methicillin-Resistance Trait

SCC*mec* typing was developed during the 2000s and has since been utilized as a valuable tool in studying the molecular epidemiology of methicillin-resistant staphylococci and investigating the evolution of various *Staphylococcus* species [31]. Molecular cloning and conventional sequencing techniques have been employed to confirm the existence and arrangement of a newly identified SCC*mec* type. In practical applications, PCR-based approaches have been widely utilized for the identification of SCC*mec*, offering convenience and efficiency over an extended period [31]. Moreover, the utilization of whole-genome sequencing has been extensively employed, leading to the recent identification of diverse SCC*mec* and analogous structures across other species [31,55]. Upon the discovery that the *mecA* gene was widely distributed across several staphylococcal species, a hypothesis emerged suggesting that *mecA* might be harbored on a MGE capable of horizontal transmission between staphylococcal species [56]. For the *mecC* gene, no study has elucidated the potential for its transfer within species of the *Staphylococcus* and *Mammaliicoccus* genera through SCC*mec* elements. 

As of now, fourteen distinct types of SCC*mec* have been documented. These types are further categorized into broad groups [31]. The size of the SCC*mec* elements varies from 21 to 82 thousand nucleotides [57]. The typical configuration of SCC*mec* cassettes encompasses five distinct sections. The categorization of SCC*mec* into distinct types is determined by the specific *ccr* chromosomal recombinase gene complex, namely *ccrA*, *ccrB*, and *ccrC* [57]. The classification of the *mec* gene complex also represents a significant factor in the division of SCC*mec*. Several distinct classes can be identified, including A, B, B2, C1, C2, D, and E. The various classes exhibit variations in the extent of *mecI-mecR* gene deletion, as well as the relative positioning and distance from the entire or truncated *IS*431, *IS*1182, and *IS*1272 [57]. The categorization of SCC*mec* subtypes is determined by the subclasses of the *mec* gene complex and the composition of the J1, J2, and J3 regions [31]. The *mec* gene complex is composed of *mecA* or *mecC*, their regulatory genes, and the accompanying insertion sequences [31]. Currently, five classes of the *mec* gene complex have been described [31]. 

### 3.2. The Mammaliicoccus Genus, a Recent Offshoot from Staphylococcus

The taxonomic characterization of *Mammaliicoccus* is derived from the existing data presented by Madhaiyan et al. [2]. The cellular composition consists of Gram-positive, nonmotile, non-spore-forming cocci, which are observed in singular form, as well as in pairs and irregular clusters. These organisms demonstrate the ability to develop under aerobic conditions, as well as under facultative anaerobic conditions. The tested samples exhibited good catalase activity, along with varying levels of oxidase activity. According to Madhaiyan et al. [2], the DNA G+C content (mol%) varies between 31.6 and 35.7, while the genome size spans from 2.44 to 2.81 Mbp. The aforementioned description pertains to *M. sciuri* comb. nov., which serves as the designated type species. The differentiation of the genus from *Staphylococcus* was achieved by the utilization of various analytical techniques, including the examination of 16S rRNA gene sequences, the construction of phylogenetic trees using whole-genome data, and the assessment of overall genome-related indices. These former *Staphylococcus* species include *M. fleurettii*, *M. lentus*, *M. sciuri*, *M. stepanovicii*, and *M. vitulinus* [2]. 

### 3.3. Ecology of mecC Gene in Non-aureus Staphylococci and Mammaliicoccus

**Figure 1:**
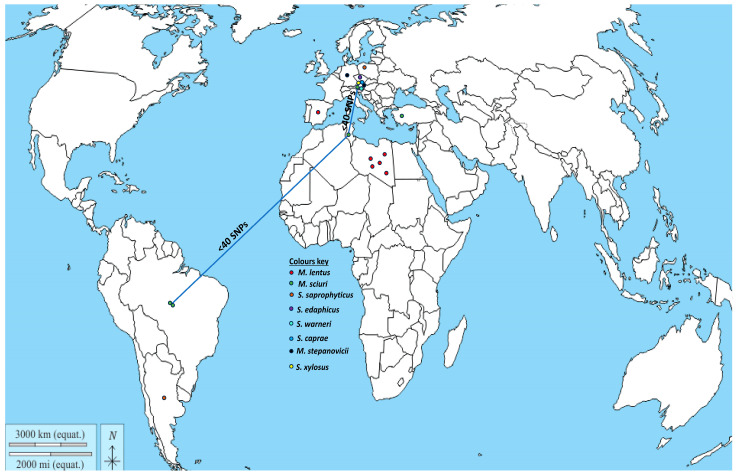
Geographical distribution of non-*aureus* staphylococci and *Mammaliicoccus* species carrying the *mecC* gene (data obtained from References [22,23,34,37,38,39,40,46,47,48,49,50,51,52,53,54]). ***NB***. The blue connecting line shows countries with genetically related *M. sciuri* strains.

The detection of the hybrid SCC*mec-mecC* in few cases in methicillin-resistant *M. sciuri* obtained in two different studies from bovine milk [34,40] indicates that the prevalence of this genetic feature in *M. sciuri* may be more extensive than previously known. Notwithstanding, the *mecC* gene has been detected in several non-*aureus* staphylococci and mammaliicocci in Europe, Africa, America, and Turkey (Figure 1); these include *M. lentus*, *S. xylosus*, *M. stepanovicii*, *S. caprae*, and *S. warneri*. Remarkably, most of these *mecC*-carrying strains were identified from dairy animals. Of the 15 studies that reported the detection of the *mecC* gene in non-*aureus* staphylococci and *Mammaliicoccus*, the most frequently identified species were *M. scuiri* and *S. xylosus.* The detection of *mecC* carrying-*M. sciuri* in both manure and milk samples suggests that contamination may have occurred due to the mammary secretions of cows suffering from mastitis [38,58]. Ecologically, *mecC*-carrying *S. xylosus* has been detected in fermented food products such as sausage [59,60] and cheese [61], thus indicating a potential pathway for the transfer of *mecC* and other resistance genes from the environment or animal product (such as bovine milk) contaminated with bacteria carrying these AMR genes [22,62].

As most *mecC*-carrying non-*aureus* staphylococci and *Mammaliicoccus* are associated with livestock, especially dairy animals, these strains could exert negative impacts on livestock’s health, production, and public health as in the case of bovine mastitis that causes a decline in quality and quantity of milk and milk product [63,64,65]. Moreover, contaminated milk may cause gastroenteritis in humans when they consume dairy products contaminated with *mecC*-carrying non-*aureus* staphylococci or *Mammaliicoccus* that elaborate virulence factors such as the *icaABCD* biofilm genes [66]. It has been shown that biofilm production could exponentially facilitate the persistence of AMR in bacteria [66]. Thus, biofilms during infections and contamination of dairy products can cause public health concerns from veterinary, food safety, and medical standpoints. 

Tracing the origin of *mecC*-carrying non-*aureus* staphylococci and mammaliicocci in dairy animals could be difficult, but it could be hypothesized that this methicillin resistance trait might have been acquired from wild animals’ secretions containing the *mecC* gene, as these hosts are the major and natural reservoirs of *mecC*-mediated MRSA [26]. Interestingly, two of the three studies on wild animals reported *mecC*-carrying *M. stepanovicii* in SCC*mec* XI. However, the other was a *mecA/mecC*-carrying *M. lentus* from a nestling stork whose parent foraged in landfills that could have been contaminated by livestock pasture and feces [37]. In this regard, genomic-based surveillance has become necessary to understand the potential transmission of *mecC* gene from MRSA to non-*aureus* staphylococci and mammaliicocci in the same micro-niches or ecosystems. 

The predominance of *M. sciuri* and *S. xylosus* may be better understood by considering their ability to adapt to various ecological environments and among them the teat canal of dairy animals [67]. The organism’s capacity to inhabit both living and nonliving surfaces is likely ascribed to its capability to form a biofilm and the existence of genes linked to ecological adaptation [66]. These bacteria have widely been recognized as nonpathogenic commensal, with a limited number of documented cases associating them with diseases. In contrast, it is important to highlight that *S. saprophyticus*, which exhibits the most closely related evolutionary lineage to *S. xylosus*, possesses considerable significance as an opportunistic pathogen [68]. Specifically, *S. saprophyticus* contracted from contaminated food has long been implicated in urinary tract infections in young teenagers [68,69]. Moreover, *M. lentus* and *M. sciuri* are considered etiological agents of exudative epidermitis with zoonotic potentials [70]. Much more recently, whole-genome data of non-*aureus* staphylococci species have led to the identification and characterization of numerous putative virulence factors [71,72,73].

The finding of a beta-lactam-resistant *S. edaphicus* strain from an antarctic environment sample showed that the *mecC* gene located between a pseudo-staphylococcus cassette chromosome *mec* (ψSCC*mec*P5085) and other SCCs implies the integration and exchange of foreign DNA [54]. It has been shown that MecC protein exhibits enhanced stability and activity at lower temperatures in comparison to the MecA protein [74]. This phenomenon may provide an evolutionary advantage in mitigating the prevalence of beta-lactam producers in arctic habitats.

### 3.4. Genetic Environment of the mecC in Staphylococcus and Mammaliicoccus Species

**Figure 2:**
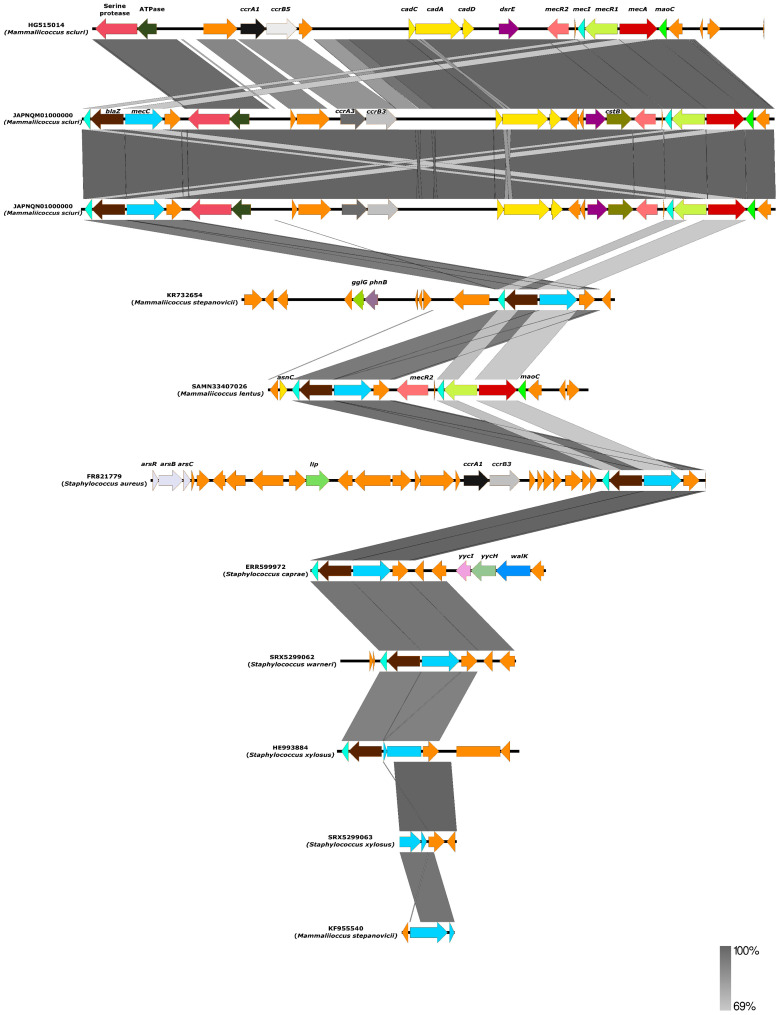
The environment of the *mecC* gene of ten non-*aureus* staphylococci and *Mammaliicoccus* species compared with previously described *S. aureus*_LGA251_ (accession number FR821779). The percentage of identity and scale bar legends are presented on the right side of the image.

From our in silico analysis of the environment of *mecC* gene of all *Mammaliicoccus* species, it appears that this gene is encoded within a hybrid SCC*mec* element comprising *mecA* encoding SCC*mec* type VII [40,47,49]. This is very different from all other *mecC*-carrying non-*aureus* staphylococci, which were all in SCC*mec* type XI (Figure 2). Specifically, the analysis of 10 *mecC-carrying* non-*aureus* staphylococci and *Mammaliicoccus* species showed that all except *S. xylosus*, *M. stepanovicii*, *S. warneri*, *S. caprae*, and *S. edaphicus* carried a hybrid SCC*mec-mecC* (Figure 2). The SCC*mec-mecC* hybrid consists of a class E *mec* complex (*mecI-mecR1-mecC1-blaZ*) located immediately downstream of a SCC*mec* type VII element (Figure 2). Most of the cassettes comprise *mecA*/*mecI*/*mecR2* and *cadD*/*cadA*/*cadC* (Figure 2). The *mecC* gene of the *S. xylosus*, *M. stepanovicii*, *S. warneri*, *S. caprae*, and *S. edaphicus* strains was very similar to SCC*mec* type XI, a classical type that was first found *in S. aureus* _LGA251_ (accession number FR821779)**.** Perhaps, this could be because only the *mecC* gene was related to the methicillin resistance in these strains. Due to the high similarity (>98%) in the environment of the *mecC* of these strains with that of the reference *S. aureus* _LGA251_, it could be hypothesized that this gene might have been transferred to the non-*aureus* staphylococci through SCC*mec* XI by horizontal gene transfer (HGT), especially as both *mecC*-MRSA and *mecC*-carrying non-*aureus* staphylococci were reported in the study of Loncaric et al. [53].

It is important to mention that using genome sequences on curated web pipelines could provide an unspecific and incorrect SCC*mec* type (in most cases SCC*mec* type III), which could be due to recombination events between the SCC*mec* type III (intrinsic for most *M. lentus* and *M. sciuri*) of the *mecA* gene and SCC*mec* type XI of the *mecC* to produce the SCC*mec-mecC* hybrid. In this regard, there is a need for caution in using PCR-based assays to detect SCC*mec* types in *mecC*-carrying mammaliicocci. Particularly, the intrinsic SCC*mec* type III or *blaZ*-SCC*mec* XI fragment in mammaliicocci could appear PCR-positive. This could be the case of the findings of Abdullahi et al. [37] and Aslantaş [48]. Thus, in silico and computational analyses of *mecA/mecC* genes from whole-genome sequences of mammaliicocci are necessary to deduce their correct SCC*mec* type. 

### 3.5. Comparison of AMR Rates in mecC-Carrying S. aureus and Non-aureus Staphylococci and Mammaliicocci

Contrary to the notion that most *mecC*-carrying MRSA present low-level AMR and rarely present an MDR phenotype, most of the *mecC*-carrying mammaliicocci presented an MDR phenotype, and AMR genes of clinical relevance. This suggests that the acquisition of other non-beta-lactam resistance genes in these strains is likely to occur with notable frequency. Specifically, many *mecC*-carrying *M. sciuri* strains exhibited the highest frequencies of resistance to erythromycin, clindamycin, tetracycline, chloramphenicol, and trimethoprim–sulfamethoxazole (Table 2). 

It is important to mention that the majority of mammaliicocci strains exhibit resistance to several clinically relevant AMRs located in plasmids and transposons, especially *tet*(L), *ant4′*, *ermB*, *str*, *fexA*, and *dfrK* genes. Moreover, the presence of *M. sciuri* strains from a sheep and a goat carrying the *cfr* gene further highlights the potential of *mecC*-carrying *M. sciuri* to carry and transmit critical AMR. It is noteworthy to remark that the *cfr* gene, responsible for encoding a methyltransferase enzyme that alters the A2503 location of the 23S ribosomal RNA, was initially identified in a calf-derived strain of *M. sciuri* in the year 2000 [75]. The *cfr* gene provides resistance to multiple classes of antibiotics, including lincosamides, streptogramin A, phenicols, linezolid, and pleuromutilins [75], especially in staphylococci [7]. 

It has been observed that *fexA* gene that encodes for chloramphenicol resistance could co-select the *cfr* gene and other linezolid resistance genes in staphylococci and mammaliicocci, especially in livestock [5,7,10,76]. This shows that the persistent use of florfenicol (a derivative of chloramphenicol) in livestock farms could have encouraged the re-emergence of *cfr*-mediated linezolid resistance in many Gram-positive bacteria [7]. Tetracycline and erythromycin are frequently employed in veterinary medicine and their usage may potentially account for the elevated rates of resistance. Contrary to these observations, all the *mecC*-carrying non-*aureus* staphylococci did not present an MDR phenotype, a feature that is closely similar to the *mecC*-carrying-MRSA. This further supports the hypothesis that *mecC*-carrying non-*aureus* staphylococci could have similar evolutionary origins of SCC*mec* type XI and low-level resistance to non-beta-lactams. 

### 3.6. Phylogenomic Relatedness of mecC-Carrying Non-aureus Staphylococci and Mammaliiococci

**Figure 3:**
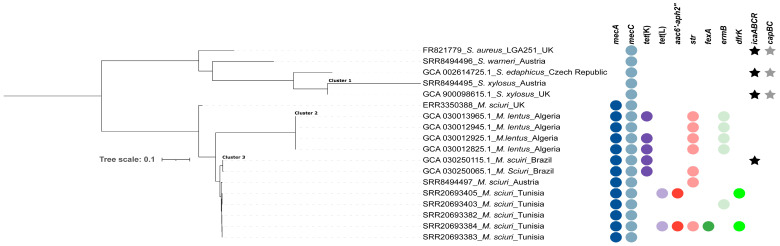
Phylogenomic tree based on core genome SNP analysis of 17 non-*aureus* staphylococci and mammaliicocci from six countries. The presence of AMR genes is indicated by filled circles, while the *icaABCR* operon and *capBC* genes are indicated by filled stars.

Mapping of the assembled genomes of the 17 *mecC*-carrying non-*aureus* staphylococci and mammaliicocci with the reference *S. aureus* _LGA251_ indicated three distinct clusters (Figure 3). Of these, two contained two *S. xylosus* strains from the UK (cluster 1), four *M. lentus* strains from Tunisia (cluster 2), and eight M. sciuri strains from Austria, Tunisia, and Brazil (cluster 3). The remaining strains (*M. sciuri*-ERR3350388, *S. warneri,* and *S. ediphicus*) existed as standalone on the tree (with wide SNP difference from other strains) (Supplementary Table S1, Figure 3). 

Analysis of a midpoint-rooted phylogenomic tree of the three clusters confirmed the close relatedness (<20 SNPs) and potential transmission of mammaliicoccal strains in livestock farms, as in the case of *M. lentus* in Algerian camels and *M. sciuri* from different types of livestock in Tunisia and Brazil (Supplementary Table S1, Figure 3). Moreover, phylogenetic analysis further showed the genetic proximity (<40 SNPs) of *M. sciuri* strains from Austria, Brazil, and Tunisia (Figure 3). These findings highlight the intercontinental circulation of related *M. sciuri* strains between various livestock species, as confirmed by the phylogenetic analysis (Figure 3). However, further studies are important to elucidate the pathway of transmission of the genetically related strains to fully understand the factors that facilitated their presence in these countries. 

## 4. Conclusions

This systematic review enhances our comprehension of the epidemiology and genetic organization of *mecC* within the non-*aureus* staphylococci and mammaliicocci. From our in silico analyses of the *mecC* gene, distinct variation in the SCC*mec* elements of non-*aureus* staphylococci from other (carrying SCC*mec-mecC*) hybrids tends to be genus-specific. Furthermore, utilizing core genome phylogenetic analysis, it was determined that the *mecA/mecC* cassette has been acquired by non-*aureus* staphylococci and mammaliicocci on separate occasions. The potential implications of clonal development of a lineage of *mecA/mecC* carrying strains across multiple dairy farms in a vast geographical region with the dissemination of the MDR phenotype is envisaged. 

It was observed that most *mecC*-carrying non-*aureus* staphylococci and mammaliicocci were detected in mastitis cases. Therefore, veterinarians and veterinary microbiology laboratories must remain vigilant regarding the potential existence of *mecA/mecC* strains originating from mastitis as a potential niche for this resistance trait.

In summary, enhancing genome-based surveillance of *mecC*-carrying non-*aureus* staphylococci and mammaliicocci is vital to ascertaining their origins and impact on human and animal health.

## Figures and Tables

**Table 1:** Beta-lactam resistance as well as mobile genetic elements carrying these genes in staphylococci, mammaliicocci, and macrococci.

Resistance Mechanism	Mobile Genetic Elements with Resistance Genes			References
	Plasmids	Transposons	Other MGEs	
a. *blaZ* (all species except *S. arlettae*)	*pI258*, *pII147*	Tn*552*, Tn*4002* and Tn*4201*	SCC*mec* type XI	García-Álvarez et al. [16], Shearer et al. [17]
b. *bla_ARL_* (only *S. arlettae*)	None	None	None	Andreis et al. [18]
c. *mecA*	None	None	Various SCC*mec* types	Miragaia [15]
i. *mecA1* (*M. sciuri*), 85% homology with *mecA*	None	None	None	Cai et al. [19]
ii. *mecA2* (*S. vitulinus*) 94% homology with *mecA*	None	None	None	Miragaia [15]
d. *mecB (S. aureus) 69% homology with mecA*	*pSAWWU4229_1*	None	None	Becker et al. [20]
e. *mecB (M. caseolyticus)*	*pMCCL2*	Tn*6045*	McRI*mecD*-1	Schwendener et al. [21]
f. *mecC* (*S. aureus* _LGA251_ and many CoNS)	None	None	SCC*mec* XI and SCC*mec-mecC* hybrids	García-Álvarez et al. [16]
i. *mecC1* gene in *S. xylosus*	None	None	SCC*mec* XI	Harrison et al. [22]
ii. *mecC2* gene in *S. saprophyticus*	None	None	SCC*mec-mecC* hybrid	Małyszko et al. [23]
g. Mutations in genes encoding PBP2 and PBP4, especially on the genes gdpP and yjbH conditioning the overproduction of PBP4 protein and resistance to ceftobiprole.	None	None	None	Greninger et al. [24] Lee et al. [25]
h. *mecD* (*Macrococcus caseolyticus*)	None	None	McRImecD-1, McRImecD-2	Schwendener et al. [21]

**Abbreviation:** SCC*mec*: staphylococcal chromosome cassette *mec*. CoNS: coagulase-negative staphylococci.

## Data Availability

Data are contained within the article and Supplementary Materials.

## Supplementary Materials

The following supporting information can be downloaded at: https://www.mdpi.com/article/10.3390/microorganisms12010066/s1, Figure S1: Identification and selection flowchart of articles on the *mecC*-carrying non-*aureus* staphylococci and mammaliicocci; Table S1: SNPs matrix of 17 genomes of *mecC*-carrying non-*aureus* staphylococci and mammaliicocci.

## References

[1] Szczuka E., Wesołowska M., Krawiec A., Kosicki J.Z. (2023). Staphylococcal species composition in the skin microbiota of domestic pigeons (Columba livia domestica). PLoS ONE.

[2] Madhaiyan M., Wirth J.S., Saravanan V.S. (2020). Phylogenomic analyses of the Staphylococcaceae family suggest the reclassification of five species within the genus *Staphylococcus* as heterotypic synonyms, the promotion of five subspecies to novel species, the taxonomic reassignment of five Staphylococcus species to Mammaliicoccus gen. nov., and the formal assignment of *Nosocomiicoccus* to the family Staphylococcaceae. Int. J. Syst. Evol. Microbiol..

[3] Sakr A., Brégeon F., Mège J.-L., Rolain J.-M., Blin O. (2018). *Staphylococcus aureus* Nasal Colonization: An Update on Mechanisms, Epidemiology, Risk Factors, and Subsequent Infections. Front. Microbiol..

[4] Raineri E.J.M., Altulea D., van Dijl J.M. (2022). Staphylococcal trafficking and infection—From ‘nose to gut’ and back. FEMS Microbiol. Rev..

[5] Gostev V., Leyn S., Kruglov A., Likholetova D., Kalinogorskaya O., Baykina M., Dmitrieva N., Grigorievskaya Z., Priputnevich T., Lyubasovskaya L. (2021). Global Expansion of Linezolid-Resistant Coagulase-Negative Staphylococci. Front. Microbiol..

[6] Abdullahi I.N., Lozano C., Simón C., Zarazaga M., Torres C. (2023). Within-Host Diversity of Coagulase-Negative Staphylococci Resistome from Healthy Pigs and Pig Farmers, with the Detection of *cfr*-Carrying Strains and MDR-*S. borealis*. Antibiotics.

[7] Brenciani A., Morroni G., Schwarz S., Giovanetti E. (2022). Oxazolidinones: Mechanisms of resistance and mobile genetic elements involved. J. Antimicrob. Chemother..

[8] Ruiz-Ripa L., Gómez P., Alonso C.A., Camacho M.C., Ramiro Y., de la Puente J., Fernández-Fernández R., Quevedo M., Blanco J.M., Báguena G. (2020). Frequency and Characterization of Antimicrobial Resistance and Virulence Genes of Coagulase-Negative Staphylococci from Wild Birds in Spain. Detection of *tst*-Carrying *S. sciuri* Isolates. Microorganisms.

[9] Al-Haqan A., Boswihi S.S., Pathan S., Udo E.E. (2022). Antimicrobial resistance and virulence determinants in coagulase-negative staphylococci isolated mainly from preterm neonates. PLoS ONE.

[10] Abdullahi I.N., Lozano C., Saidenberg A.B.S., Latorre-Fernández J., Zarazaga M., Torres C. (2023). Comparative review of the nasal carriage and genetic characteristics of *Staphylococcus aureus* in healthy livestock: Insight into zoonotic and anthroponotic clones. Infect. Genet. Evol..

[11] (2023). A One Health Priority Research Agenda for Antimicrobial Resistance. Geneva: World Health Organization, Food and Agriculture Organization of the United Nations, United Nations Environment Programme and World Organisation for Animal Health. https://www.who.int/publications-detail-redirect/9789240075924.

[12] Jori F., Hernandez-Jover M., Magouras I., Dürr S., Brookes V.J. (2021). Wildlife–livestock interactions in animal production systems: What are the biosecurity and health implications?. Anim. Front. Rev. Mag. Anim. Agric..

[13] Lee S., Fan P., Liu T., Yang A., Boughton R.K., Pepin K.M., Miller R.S., Jeong K.C. (2022). Transmission of antibiotic resistance at the wildlife-livestock interface. Commun. Biol..

[14] Ambade S.S., Gupta V.K., Bhole R.P., Khedekar P.B., Chikhale R.V. (2023). A Review on Five and Six-Membered Heterocyclic Compounds Targeting the Penicillin-Binding Protein 2 (PBP2A) of Methicillin-Resistant *Staphylococcus aureus* (MRSA). Molecules.

[15] Miragaia M. (2018). Factors Contributing to the Evolution of *mecA*-Mediated β-lactam Resistance in Staphylococci: Update and New Insights From Whole Genome Sequencing (WGS). Front. Microbiol..

[16] García-Álvarez L., Holden M.T., Lindsay H., Webb C.R., Brown D.F., Curran M.D., Walpole E., Brooks K., Pickard D.J., Teale C. (2011). Meticillin-resistant *Staphylococcus aureus* with a novel mecA homologue in human and bovine populations in the UK and Denmark: A descriptive study. Lancet Infect. Dis..

[17] Shearer J.E.S., Wireman J., Hostetler J., Forberger H., Borman J., Gill J., Sanchez S., Mankin A., LaMarre J., Lindsay J.A. (2011). Major families of multiresistant plasmids from geographically and epidemiologically diverse staphylococci. G3 Genes|Genomes|Genet..

[18] Andreis S.N., Perreten V., Schwendener S. (2017). Novel β-Lactamase *bla*_ARL_ in *Staphylococcus arlettae*. mSphere.

[19] Cai Y., Zheng L., Lu Y., Zhao X., Sun Y., Tang X., Xiao J., Wang C., Tong C., Zhao L. (2021). Inducible Resistance to β-Lactams in Oxacillin-Susceptible mecA1-Positive *Staphylococcus sciuri* Isolated from Retail Pork. Front. Microbiol..

[20] Becker K., van Alen S., Idelevich E.A., Schleimer N., Seggewiß J., Mellmann A., Kaspar U., Peters G. (2018). Plasmid-Encoded Transferable *mecB*-Mediated Methicillin Resistance in *Staphylococcus aureus*. Emerg. Infect. Dis..

[21] Schwendener S., Cotting K., Perreten V. (2017). Novel methicillin resistance gene *mecD* in clinical *Macrococcus caseolyticus* strains from bovine and canine sources. Sci. Rep..

[22] Harrison E.M., Paterson G.K., Holden M.T.G., Morgan F.J.E., Larsen A.R., Petersen A., Leroy S., De Vliegher S., Perreten V., Fox L.K. (2013). A *Staphylococcus xylosus* isolate with a new *mecC* allotype. Antimicrob. Agents Chemother..

[23] Yszko I.M., Schwarz S., Hauschild T. (2014). Detection of a new *mecC* allotype, *mecC2*, in methicillin-resistant *Staphylococcus saprophyticus*. J. Antimicrob. Chemother..

[24] Greninger A.L., Chatterjee S.S., Chan L.C., Hamilton S.M., Chambers H.F., Chiu C.Y. (2016). Whole-Genome Sequencing of Methicillin-Resistant *Staphylococcus aureus* Resistant to Fifth-Generation Cephalosporins Reveals Potential Non-mecA Mechanisms of Resistance. PLoS ONE.

[25] Lee H., Yoon E.-J., Kim D., Kim J.W., Lee K.-J., Kim H.S., Kim Y.R., Shin J.H., Shin J.H., Shin K.S. (2018). Ceftaroline Resistance by Clone-Specific Polymorphism in Penicillin-Binding Protein 2a of Methicillin-Resistant *Staphylococcus aureus*. Antimicrob. Agents Chemother..

[26] Abdullahi I.N., Fernández-Fernández R., Juárez-Fernández G., Martínez-Álvarez S., Eguizábal P., Zarazaga M., Lozano C., Torres C. (2021). Wild Animals Are Reservoirs and Sentinels of *Staphylococcus aureus* and MRSA Clones: A Problem with “One Health” Concern. Antibiotics.

[27] Abdullahi I.N., Lozano C., Ruiz-Ripa L., Fernández-Fernández R., Zarazaga M., Torres C. (2021). Ecology and Genetic Lineages of Nasal *Staphylococcus aureus* and MRSA Carriage in Healthy Persons with or without Animal-Related Occupational Risks of Colonization: A Review of Global Reports. Pathogens.

[28] Shore A.C., Deasy E.C., Slickers P., Brennan G., O’Connell B., Monecke S., Ehricht R., Coleman D.C. (2011). Detection of staphylococcal cassette chromosome *mec* type XI carrying highly divergent *mecA*, *mecI*, *mecR1*, *blaZ*, and *ccr* genes in human clinical isolates of clonal complex 130 methicillin-resistant *Staphylococcus aureus*. Antimicrob. Agents Chemother..

[29] Paterson G.K., Larsen A.R., Robb A., Edwards G.E., Pennycott T.W., Foster G., Mot D., Hermans K., Baert K., Peacock S.J. (2012). The newly described mecA homologue, mecALGA251, is present in methicillin-resistant *Staphylococcus aureus* isolates from a diverse range of host species. J. Antimicrob. Chemother..

[30] Lakhundi S., Zhang K. (2018). Methicillin-Resistant *Staphylococcus aureus*: Molecular Characterization, Evolution, and Epidemiology. Clin. Microbiol. Rev..

[31] Uehara Y. (2022). Current Status of Staphylococcal Cassette Chromosome *mec* (SCC*mec*). Antibiotics.

[32] Lozano C., Fernández-Fernández R., Ruiz-Ripa L., Gómez P., Zarazaga M., Torres C. (2022). Human *mecC*-Carrying MRSA: Clinical Implications and Risk Factors. Microorganisms.

[33] Gómez P., Ruiz-Ripa L., Fernández-Fernández R., Gharsa H., Ben Slama K., Höfle U., Zarazaga M., Holmes M.A., Torres C. (2021). Genomic Analysis of *Staphylococcus aureus* of the Lineage CC130, Including *mecC*-Carrying MRSA and MSSA Isolates Recovered of Animal, Human, and Environmental Origins. Front. Microbiol..

[34] Harrison E.M., Paterson G.K., Holden M.T.G., Ba X., Rolo J., Morgan F.J.E., Pichon B., Kearns A., Zadoks R.N., Peacock S.J. (2014). A novel hybrid *SCCmec-mecC* region in *Staphylococcus sciuri*. J. Antimicrob. Chemother..

[35] Rolo J., de Lencastre H., Miragaia M. (2014). High frequency and diversity of cassette chromosome recombinases (*ccr*) in methicillin-susceptible *Staphylococcus sciuri*. J. Antimicrob. Chemother..

[36] Rolo J., Worning P., Nielsen J.B., Bowden R., Bouchami O., Damborg P., Guardabassi L., Perreten V., Tomasz A., Westh H. (2017). Evolutionary Origin of the Staphylococcal Cassette Chromosome *mec* (SCC *mec*). Antimicrob. Agents Chemother..

[37] Abdullahi I.N., Lozano C., Höfle Ú., Cardona-Cabrera T., Zarazaga M., Torres C. (2023). Antimicrobial resistome of coagulase-negative staphylococci from nasotracheal cavities of nestlings of *Ciconia ciconia* in Southern Spain: Detection of *mecC*-*SCCmec type*-XI-carrying *S. lentus*. Comp. Immunol. Microbiol. Infect. Dis..

[38] Dhaouadi S., Soufi L., Campanile F., Dhaouadi F., Sociale M., Lazzaro L., Cherif A., Stefani S., Elandoulsi R.B. (2022). Prevalence of meticillin-resistant and -susceptible coagulase-negative staphylococci with the first detection of the *mecC* gene among cows, humans and manure in Tunisia. Int. J. Antimicrob. Agents.

[39] Dhaouadi S., Bouchami O., Soufi L., Dhaouadi F., Chaari S., Bouglita W., Cherif A., de Lencastre H., Elandoulsi R.B., Miragaia M. (2022). Frequent dissemination and carriage of an SCC*mec*-*mecC* hybrid in methicillin-resistant *Mammaliicoccus sciuri* in farm animals from Tunisia. J. Glob. Antimicrob. Resist..

[40] Paterson G.K. (2020). Genomic epidemiology of methicillin-resistant *Staphylococcus sciuri* carrying a *SCCmec-mecC* hybrid element. Infect. Genet. Evol..

[41] Coimbra D.G., Almeida A.G.C.S., Jùnior J.B.O., Silva L.A.F.D., Pimentel B.J., Gitaí D., Moreira L.S., Silva-Filho E.A., de Andrade T. (2011). Wound infection by multiresistant *Staphylococcus sciuri* identified by molecular methods. New Microbiol..

[42] Nemeghaire S., Argudín M.A., Feßler A.T., Hauschild T., Schwarz S., Butaye P. (2014). The ecological importance of the *Staphylococcus sciuri* species group as a reservoir for resistance and virulence genes. Vet. Microbiol..

[43] Grazul M., Balcerczak E., Sienkiewicz M. (2023). Analysis of the Presence of the Virulence and Regulation Genes from *Staphylococcus aureus* (*S. aureus*) in Coagulase Negative Staphylococci and the Influence of the Staphylococcal Cross-Talk on Their Functions. Int. J. Environ. Res. Public Health.

[44] Letunic I., Bork P. (2021). Interactive Tree Of Life (iTOL) v5: An online tool for phylogenetic tree display and annotation. Nucleic Acids Res..

[45] Jolley K.A., Bray J.E., Maiden M.C.J. (2018). Open-access bacterial population genomics: BIGSdb software, the PubMLST.org website and their applications. Wellcome Open Res..

[46] MacFadyen A.C., Harrison E.M., Ellington M.J., Parkhill J., Holmes M.A., Paterson G.K. (2018). A highly conserved *mecC*-encoding SCC*mec*type XI in a bovine isolate of methicillin-resistant *Staphylococcus xylosus*. J. Antimicrob. Chemother..

[47] de Moura G.S., de Carvalho E., Sanchez E.M.R., Sellera F.P., Marques M.F., Heinemann M.B., De Vliegher S., Souza F.N., Mota R.A. (2023). Emergence of livestock-associated *Mammaliicoccus sciuri* ST71 co-harbouring *mecA* and *mecC* genes in Brazil. Vet. Microbiol..

[48] Aslantas O. (2020). High Occurence of Methicillin Resistant *Staphylococcus sciuri* (MRSS) and First Detection of *mecC* from Broiler Flocks in Turkey. Isr. J. Vet. Med..

[49] Belhout C., Boyen F., Vereecke N., Theuns S., Taibi N., Stegger M., de la Fé-Rodríguez P.Y., Bouayad L., Elgroud R., Butaye P. (2023). Prevalence and Molecular Characterization of Methicillin-Resistant Staphylococci (MRS) and Mammaliicocci (MRM) in Dromedary Camels from Algeria: First Detection of SCC*mec*-*mecC* Hybrid in Methicillin-Resistant *Mammaliicoccus lentus*. Antibiotics.

[50] Srednik M.E., Archambault M., Jacques M., Gentilini E.R. (2017). Detection of a mecC-positive *Staphylococcus saprophyticus* from bovine mastitis in Argentina. J. Glob. Antimicrob. Resist..

[51] Loncaric I., Kübber-Heiss A., Posautz A., Stalder G.L., Hoffmann D., Rosengarten R., Walzer C. (2013). Characterization of methicillin-resistant *Staphylococcus* spp. carrying the *mecC* gene, isolated from wildlife. J. Antimicrob. Chemother..

[52] Semmler T., Harrison E.M., Lübke-Becker A., Ulrich R.G., Wieler L.H., Guenther S., Stamm I., Hanssen A.-M., Holmes M.A., Vincze S. (2016). A Look into the Melting Pot: The *mecC*-Harboring Region Is a Recombination Hot Spot in *Staphylococcus stepanovicii*. PLoS ONE.

[53] Loncaric I., Kübber-Heiss A., Posautz A., Ruppitsch W., Lepuschitz S., Schauer B., Feßler A.T., Krametter-Frötscher R., Harrison E.M., Holmes M.A. (2019). Characterization of mecC gene-carrying coagulase-negative *Staphylococcus* spp. isolated from various animals. Vet. Microbiol..

[54] Pantůček R., Sedláček I., Indráková A., Vrbovská V., Mašlaňová I., Kovařovic V., Švec P., Králová S., Krištofová L., Kekláková J. (2018). *Staphylococcus edaphicus* sp. nov., Isolated in Antarctica, Harbors the *mecC* Gene and Genomic Islands with a Suspected Role in Adaptation to Extreme Environments. Appl. Environ. Microbiol..

[55] Kaya H., Hasman H., Larsen J., Stegger M., Johannesen T.B., Allesøe R.L., Lemvigh C.K., Aarestrup F.M., Lund O., Larsen A.R. (2018). SCC *mec* Finder, a Web-Based Tool for Typing of Staphylococcal Cassette Chromosome *mec* in *Staphylococcus aureus* Using Whole-Genome Sequence Data. mSphere.

[56] Wielders C.L.C., Fluit A.C., Brisse S., Verhoef J., Schmitz F.J. (2002). *mecA* gene is widely disseminated in *Staphylococcus aureus* population. J. Clin. Microbiol..

[57] Mlynarczyk-Bonikowska B., Kowalewski C., Krolak-Ulinska A., Marusza W. (2022). Molecular Mechanisms of Drug Resistance in *Staphylococcus aureus*. Int. J. Mol. Sci..

[58] Cobirka M., Tancin V., Slama P. (2020). Epidemiology and Classification of Mastitis. Animals.

[59] Leroy S., Christieans S., Talon R. (2019). Tetracycline Gene Transfer in *Staphylococcus xylosus in situ* during Sausage Fermentation. Front. Microbiol..

[60] Leroy S., Vermassen A., Ras G., Talon R. (2017). Insight into the Genome of *Staphylococcus xylosus*, a Ubiquitous Species Well Adapted to Meat Products. Microorganisms.

[61] Leroy S., Even S., Micheau P., De La Foye A., Laroute V., Le Loir Y., Talon R. (2020). Transcriptomic Analysis of *Staphylococcus xylosus* in Solid Dairy Matrix Reveals an Aerobic Lifestyle Adapted to Rind. Microorganisms.

[62] Koutsoumanis K., Allende A., Álvarez-Ordóñez A., Bolton D., Bover-Cid S., Chemaly M., Davies R., De Cesare A., Herman L., EFSA Panel on Biological Hazards (BIOHAZ) (2022). Transmission of antimicrobial resistance (AMR) during animal transport. EFSA J..

[63] Paramasivam R., Gopal D.R., Dhandapani R., Subbarayalu R., Elangovan M.P., Prabhu B., Veerappan V., Nandheeswaran A., Paramasivam S., Muthupandian S. (2023). Is AMR in Dairy Products a Threat to Human Health? An Updated Review on the Origin, Prevention, Treatment, and Economic Impacts of Subclinical Mastitis. Infect. Drug Resist..

[64] Sharun K., Dhama K., Tiwari R., Gugjoo M.B., Yatoo M.I., Patel S.K., Pathak M., Karthik K., Khurana S.K., Singh R. (2021). Advances in therapeutic and managemental approaches of bovine mastitis: A comprehensive review. Vet. Q..

[65] Cheng W.N., Han S.G. (2022). Bovine mastitis: Risk factors, therapeutic strategies, and alternative treatments—A review. Asian-Australas. J. Anim. Sci..

[66] França A., Gaio V., Lopes N., Melo L.D.R. (2021). Virulence Factors in Coagulase-Negative Staphylococci. Pathogens.

[67] Traversari J., Borne B.H.P.v.D., Dolder C., Thomann A., Perreten V., Bodmer M. (2019). Non-*aureus* Staphylococci Species in the Teat Canal and Milk in Four Commercial Swiss Dairy Herds. Front. Vet. Sci..

[68] Lawal O.U., Fraqueza M.J., Bouchami O., Worning P., Bartels M.D., Gonçalves M.L., Paixão P., Gonçalves E., Toscano C., Empel J. (2021). Foodborne Origin and Local and Global Spread of *Staphylococcus saprophyticus* Causing Human Urinary Tract Infections. Emerg. Infect. Dis..

[69] Lawal O.U., Fraqueza M.J., Worning P., Bouchami O., Bartels M.D., Goncalves L., Paixão P., Goncalves E., Toscano C., Empel J. (2021). *Staphylococcus saprophyticus* Causing Infections in Humans Is Associated with High Resistance to Heavy Metals. Antimicrob. Agents Chemother..

[70] Kalai S., Roychoudhury P., Dutta T., Subudhi P., Chakraborty S., Barman N., Sen A. (2021). Multidrug resistant staphylococci isolated from pigs with exudative epidermitis in North eastern Region of India. Lett. Appl. Microbiol..

[71] Lin S., Sun B., Shi X., Xu Y., Gu Y., Gu X., Ma X., Wan T., Xu J., Su J. (2021). Comparative Genomic and Pan-Genomic Characterization of *Staphylococcus epidermidis* from Different Sources Unveils the Molecular Basis and Potential Biomarkers of Pathogenic Strains. Front. Microbiol..

[72] Ayala D.I., Grum D.S., Evans N.P., Russo K.N., Kimminau E.A., Trible B.R., Lahoti M.M., Novak C.L., Karnezos T.P. (2023). Identification and characterization of the causative agents of Focal Ulcerative Dermatitis in commercial laying hens. Front. Vet. Sci..

[73] Argemi X., Hansmann Y., Prola K., Prévost G. (2019). Coagulase-Negative Staphylococci Pathogenomics. Int. J. Mol. Sci..

[74] Kim C., Milheiriço C., Gardete S., Holmes M.A., Holden M.T.G., de Lencastre H., Tomasz A. (2012). Properties of a novel PBP2A protein homolog from *Staphylococcus aureus* strain LGA251 and its contribution to the β-lactam-resistant phenotype. J. Biol. Chem..

[75] Long K.S., Poehlsgaard J., Kehrenberg C., Schwarz S., Vester B. (2006). The Cfr rRNA methyltransferase confers resistance to Phenicols, Lincosamides, Oxazolidinones, Pleuromutilins, and Streptogramin A antibiotics. Antimicrob. Agents Chemother..

[76] Schouls L.M., Veldman K., Brouwer M.S.M., Dierikx C., Witteveen S., van Santen-Verheuvel M., Hendrickx A.P.A., Landman F., Hengeveld P., Wullings B. (2020). *cfr* and *fexA* genes in methicillin-resistant *Staphylococcus aureus* from humans and livestock in the Netherlands. Commun. Med..

