# Supplementary material for: Beyond the Wild MRSA: Genetic Features and Phylogenomic Review of mecC-Mediated Methicillin Resistance in Non-aureus Staphylococci and Mammaliicocci
Source: Microorganisms. 2023 Dec 29;12(1):66. doi: 10.3390/microorganisms12010066 (PMC10818522; doi:10.3390/microorganisms12010066)
Supplement: Supplementary file 1 [file microorganisms-12-00066-s001.zip › Supplementary Figure S1. Identification and selection flowchart of articles on the mecC-carrying non-aureus staphylococci and mammaliicocci (1).pdf]

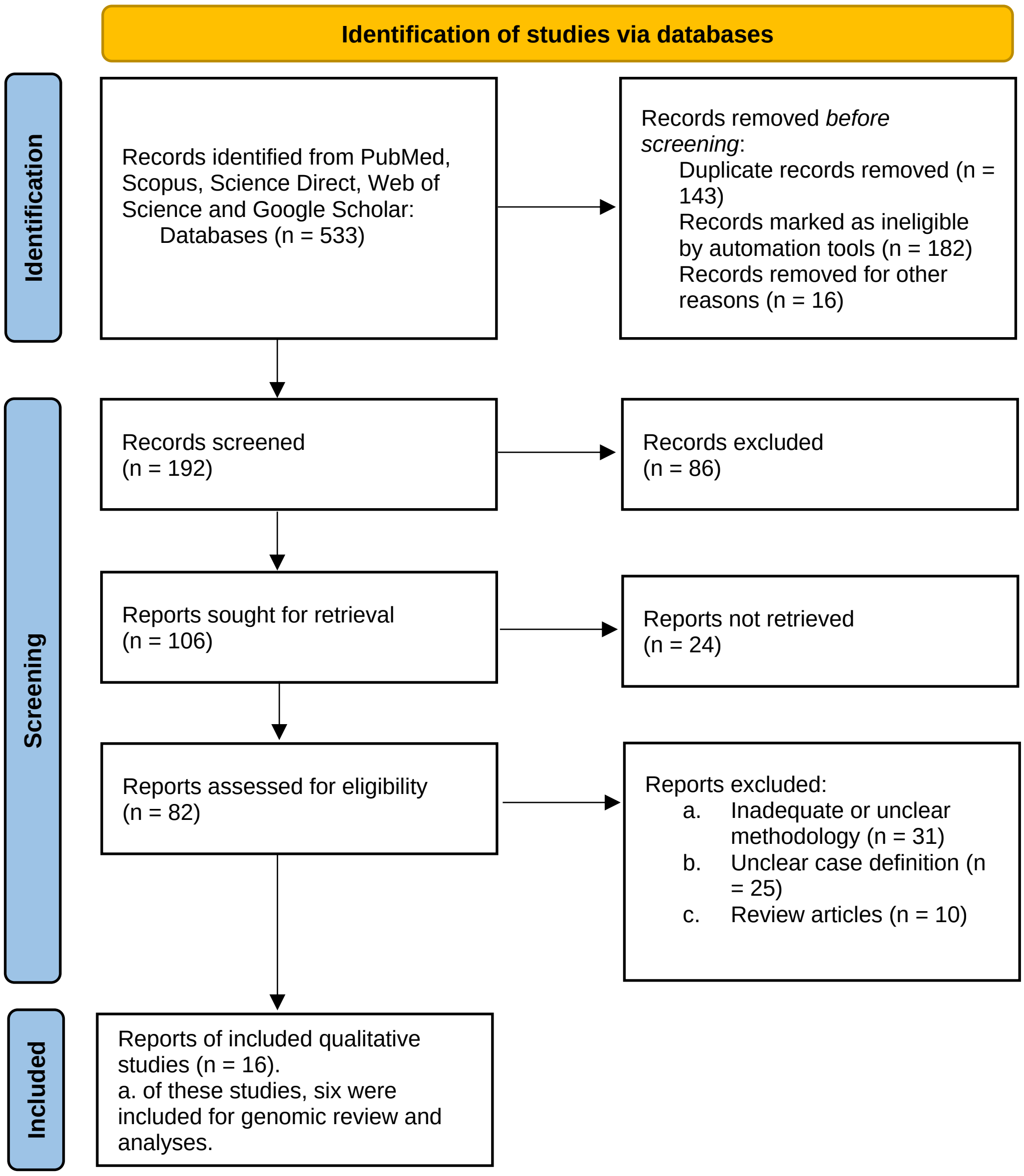

Figure S1. Identification and selection flowchart of articles on the *mecC*-carrying non-*aureus* staphylococci and mammaliicocci
